# Studying targeted oxidation in diabetic cognitive dysfunction based on scientometrics analysis: research progress of natural product approaches

**DOI:** 10.3389/fendo.2024.1445750

**Published:** 2024-12-20

**Authors:** Wenling Tu, Fuhang Xu, Jieying Li, Xiangfeng Tian, Lingyong Cao, Lei Wang, Yiqian Qu

**Affiliations:** School of Basic Medical Sciences, Zhejiang Chinese Medical University, Hangzhou, China

**Keywords:** diabetic cognitive dysfunction, scientometrics analysis, oxidative stress, natural product, Bibliometrix, VOSviewer

## Abstract

**Purpose:**

The aim is to provide new insights for researchers studying the pathogenesis of diabetic cognitive dysfunction and promoting the wider use of natural products in their treatment.

**Method:**

First, the Web of Science Core Collection was selected as the data source for a computerized literature search on oxidative stress and diabetic cognitive dysfunction (DCD). Next, Biblimetrix and VOSviewer performed statistical analysis focusing on publication countries, institutions, authors, research hotspots, and emerging directions in the field. Then, through the analysis of keywords and key articles, the forefront of the field is identified. Finally, we discussed the pathogenesis of DCD, the influence of oxidative stress on DCD and the antioxidant effect of natural products on DCD.

**Result:**

293 valid papers were obtained. Bibliometrics showed that oxidative stress, diabetes, Alzheimer’s disease (AD), cognitive decline, insulin resistance and quercetin were the key words of the symbiotic network.

**Conclusion:**

The antioxidant effects of natural products in improving DCD have been extensively studied in preclinical studies, providing potential for their treatment in DCD, but their evaluation in clinical trials is currently uncommon.

## Graphical Abstract

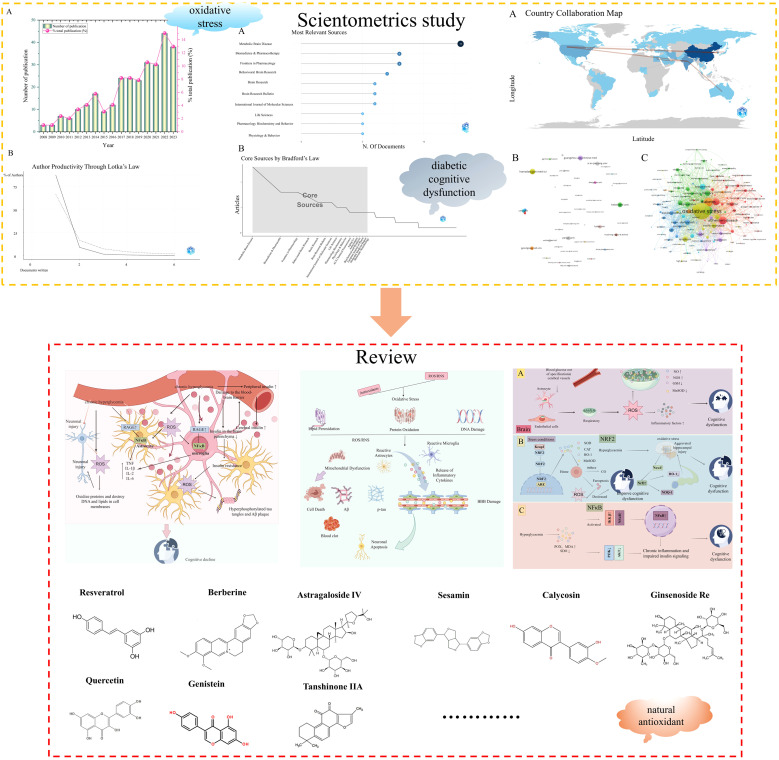

## 1 Introduction

Diabetes is a common chronic disease with many complications, and its main characteristic is high glucose concentration in the blood, which is caused by absolute or relative insulin secretion insufficiency and/or insulin utilization disorder (1, 2). The study found that diabetes can damage brain structure and function, and people with diabetes are 1.14 times more likely to develop cognitive impairment (3, 4). Diabetic cognitive dysfunction (DCD) is caused by direct neuronal injury, decreased insulin secretion, neuroinflammation, oxidative stress, and other factors caused by persistent hyperglycemia, which is manifested as impaired overall memory, attention, reasoning ability, and learning ability of patients (5, 6). With the increasing incidence of diabetes worldwide, DCD has emerged as a serious medical and social problem.

Oxidative stress is brought about by means of immoderate manufacturing and/or much less clearance of reactive oxygen species (ROS) due to endogenous or exogenous stimuli, ensuing in an imbalance of oxidation-antioxidant homeostasis and inflicting oxidative harm in tissue cells (7). Hyperglycemia is one of the typical features of DCD, which can promote the overexpression of lipid peroxides (LPO) and ROS, induce central oxidative stress to cause brain injury (8, 9). As we all know, ROS is a neurotoxic agent, which can not only cause neuronal damage and death through oxidizing proteins and destroying DNA and lipids in cell membranes but also cause neuroinflammation by promoting the secretion of inflammatory factors, ultimately leading to cognitive decline (10–12). Although it is an interesting and meaningful study to investigate the mechanism of oxidative stress on DCD, no systematic review on the association between oxidative stress and DCD has been conducted to date. Therefore, scientometrics analysis of publications in this field is necessary, and it is worth noting that scientometrics analysis is an effective method to comprehensively review this field.

Scientometrics is an emerging discipline based on various literature and integrating mathematics, computer science and statistics to study the laws, scientific management and characteristics of literature. It was named by Professor Pritchard in 1969. Scientometrics has outstanding quantitative and practical characteristics, both in terms of theoretical research. In practice, it is presented in the form of a graph based on a certain amount of data, which belongs to a type of data mining (13). Scientometrics research can analyze the progress of research in specific fields from three perspectives: 1. Quantitative analysis of basic citation, such as citation frequency analysis, coupling analysis, etc. 2. Quantitative analysis of authors, such as publication frequency, citation times, etc. 3. Quantitative analysis of vocabulary, such as keyword co-occurrence analysis, keyword distribution according to time, etc. (14), which enables researchers to rapidly and deeply discover the subject evolution and new research directions in specific research fields (15). Therefore, we conducted a scientometrics analysis of literature related to oxidative stress and DCD to explore research hotspots and tendencies, which will serve as a practical basis for later research.

## 2 Scientometrics study

### 2.1 Data and methods

#### 2.1.1 Data search, filtering, and collection

On December 18, 2023, studies related to oxidative stress in DCD were obtained using the core database of Web of Science. To search for oxidative stress-related DCD studies, we employed the following search terms: TS= (“diabetic cognitive impairment” or “diabetic cognitive dysfunction”) AND TS=oxidative stress. The language we searched was limited to English. Only two literature types were selected, articles and reviews, others were excluded.

#### 2.1.2 Statistical analysis

Initially, Origin software was employed to generate bar and line charts illustrating the annual publication counts. Secondly, VOSviewer (version 1.6.18) was employed to identify high-yielding countries, institutions, journals, authors, most cited literature, and keywords, and to conduct network mapping. Thirdly, the R package “Bibliometrix” software was utilized to analyze the data and generate correlation maps, including author productivity according to Lotka’s Law, the most relevant sources, core sources based on Bradford’s Law, and a map of country collaborations.

### 2.2 General analysis

**Figure 1:**
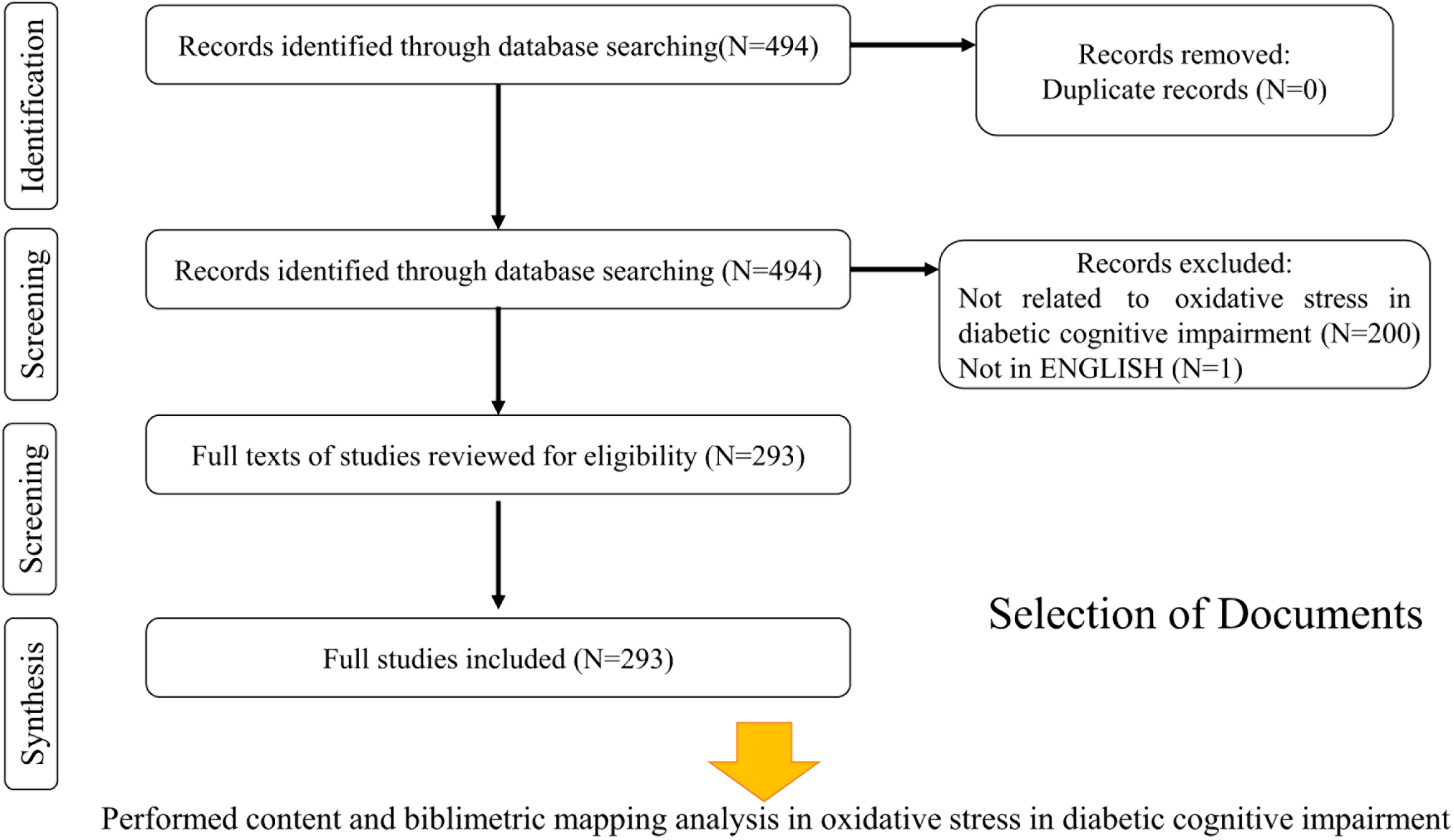
The steps of data filtering in oxidative stress research in DCD.

**Figure 2:**
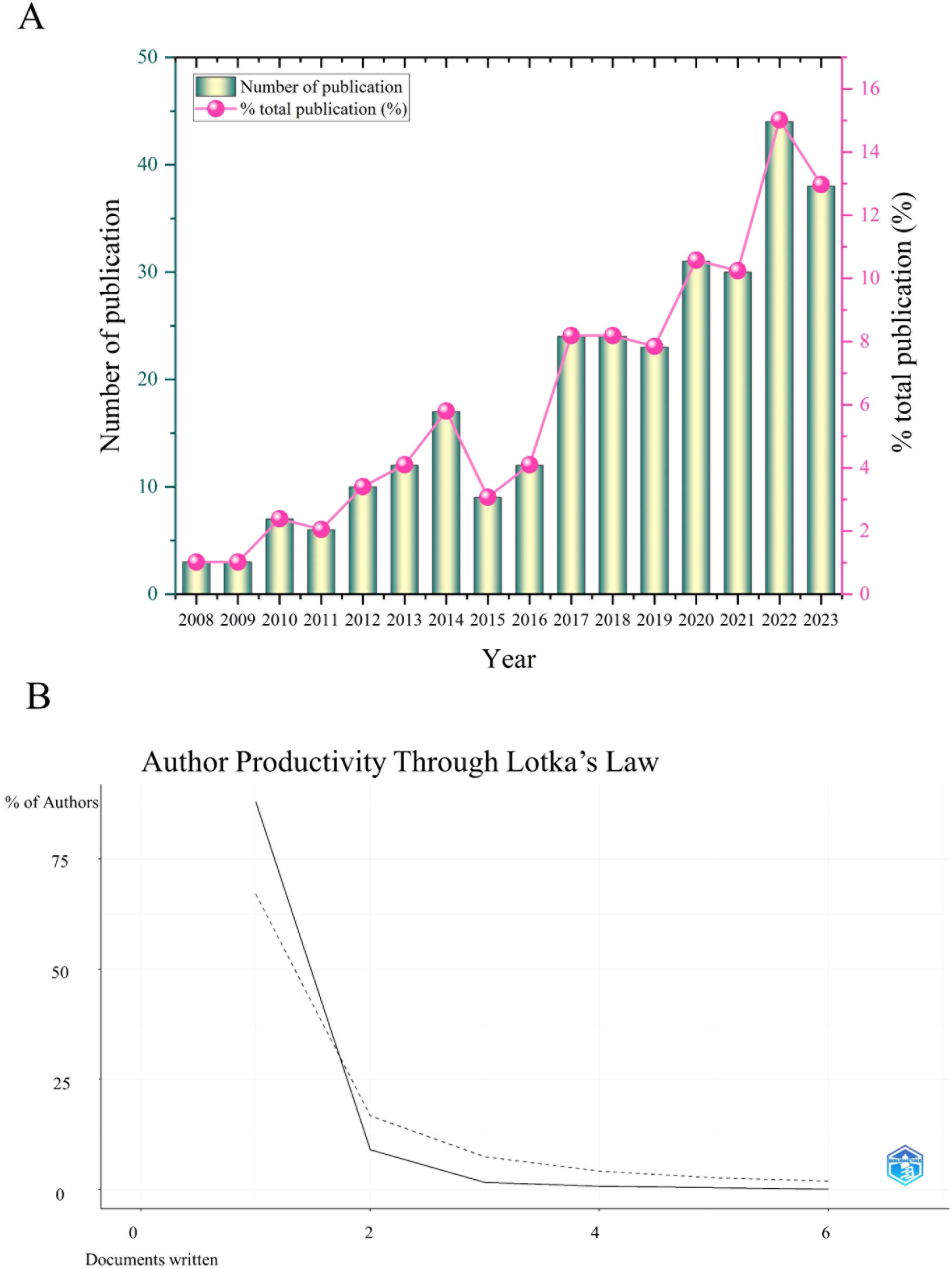
Number of articles published related to oxidative stress in DCD per year **(A)** and the author’s productivity through Lotka’s law related to oxidative stress in DCD **(B)**.

A total of 494 publications related to oxidative stress in DCD were retrieved from the core Web of Science database, comprising 401 articles and 93 reviews. Finally, 293 valid articles were obtained through the search articles cleaned for data. **Figure 1** shows the steps of data filtering. Starting in 2008, the annual number of publications increased from 3 to 44 (**Figure 2A**).

### 2.3 Author analysis

Price argues that half of all publications on the same theme are authored by high-productivity authors, with the number of these authors being equal to the square root of all scientific authors (16), that is:


$$\text{m}=0.749\times \sqrt{{\text{n}}_{\max}}$$

n_max_ indicates the number of publications the author of the most published articles. According to the results in **Table 1**: n_max_=5. m = 0.749× $\sqrt{5}$ ≈ 1.67, so authors with two or more publications are positioned as core authors in this field (**Figure 2B**). **Table 1** shows authors who have published four or more papers on this subject. From the published paper analysis, the top two scholars are more concerned that natural products can reduce cognitive deficits in DCD models by improving oxidative stress.

**Table 1:** Top 10 productive authors oxidative stress in research in DCD.

Rank	Author	Articles	Citations	Average Citation/Publication
1	Chopra,Kanwaljit	5	404	80.80
2	Baluchnejadmojarad, Tourandokht	5	249	49.80
3	Roghani,Mehrdad	5	249	49.80
4	Chen, Yun-bo	5	158	31.60
5	Wang, Qi	5	158	31.60
6	Zhang, Shi-jie	5	158	31.60
7	Kuhad, Anuray	4	351	87.75
8	Shahidi, Siamak	4	190	47.50
9	Heo, Hojin	4	36	9.00
10	Kang, Jinyong	4	36	9.00

### 2.4 Journal analysis

**Figure 3:**
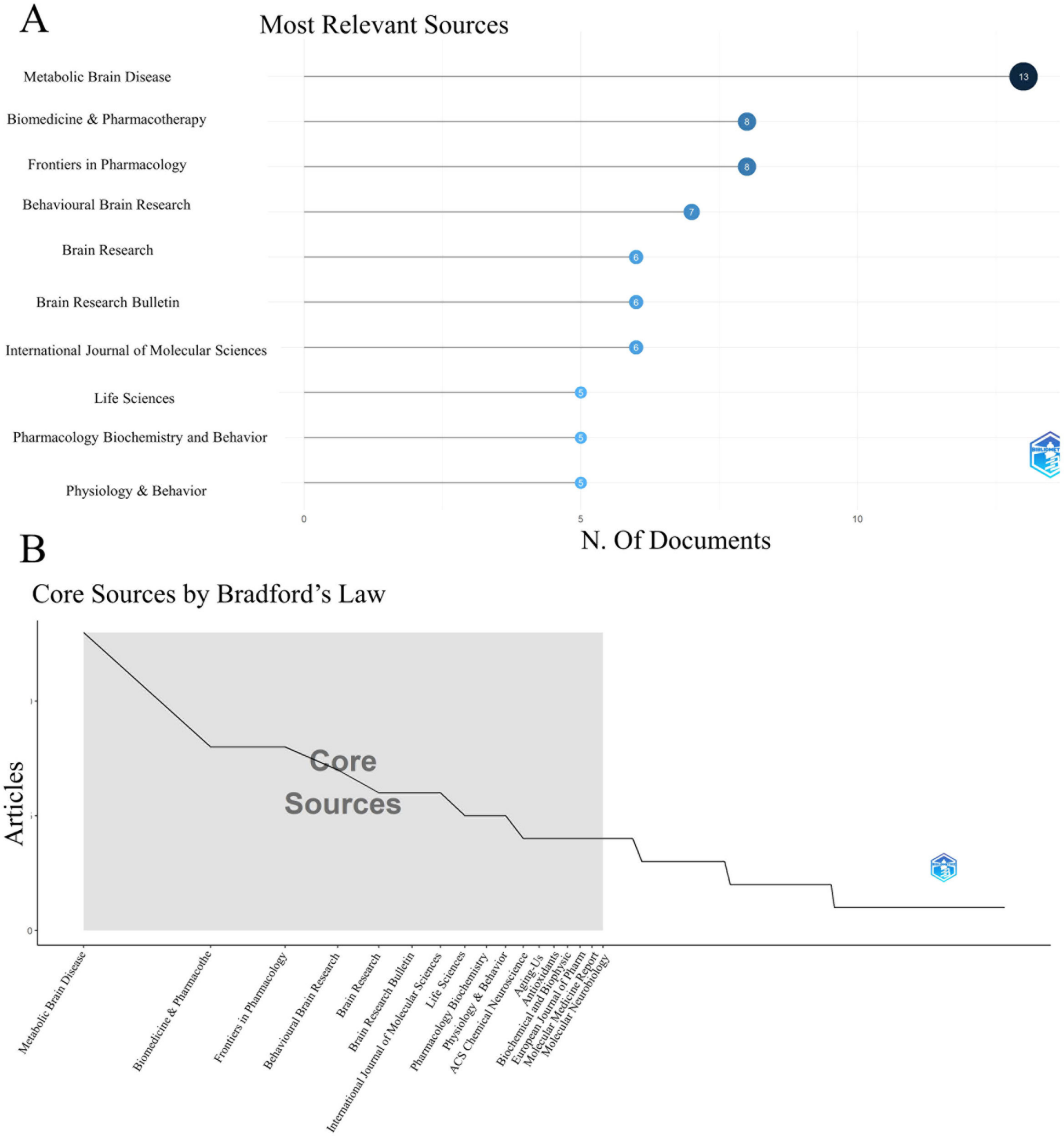
Top 10 most relevant sources for oxidative stress research in DCD **(A)** and the core sources by Bradford’s Law for oxidative stress research in DCD **(B)**.

**Table 2** and **Figure 3A** present the top ten journals based on the volume of articles. Among these, the journals featuring seven or more articles included Metabolic Brain Disease, Biomedicine & Pharmacotherapy, Frontiers in Pharmacology, and Behavioral Brain Research, with 13, 8, 8, and 7 articles, respectively. Bradford’s law is an effective law that reveals the distribution of literature information in journals. Bradford’s law is used to arrange periodicals in descending order according to the number of publications, and the core region, relevant region, and discrete region can be calculated. According to the results of **Figure 3B**, we found that there are 17 journals located in the core area, of which Metabolic Brain Diseases and Biomedical & Pharmacotherapy are the top two core journals in this field.

**Table 2:** Top 10 most relevant sources for oxidative stress research in DCD.

Rank	Sources	Articles	Citations	Average Citation/Publication
1	METABOLIC BRAIN DISEASE	13	208	16.00
2	BIOMEDICINE & PHARMACOTHERAPY	8	268	33.50
3	FRONTIERS IN PHARMACOLOGY	8	169	21.13
4	BEHAVIOURAL BRAIN RESEARCH	7	461	65.86
5	BRAIN RESEARCH	6	167	27.83
6	BRAIN RESEARCH BULLETIN	6	159	26.50
7	INTERNATIONAL JOURNAL OF MOLECULAR SCIENCES	6	175	29.17
8	LIFE SCIENCES	5	231	46.20
9	PHARMACOLOGY BIOCHEMISTRY AND BEHAVIOR	5	351	70.20
10	PHYSIOLOGY & BEHAVIOR	5	196	39.20

### 2.5 Country analysis

**Figure 4:**
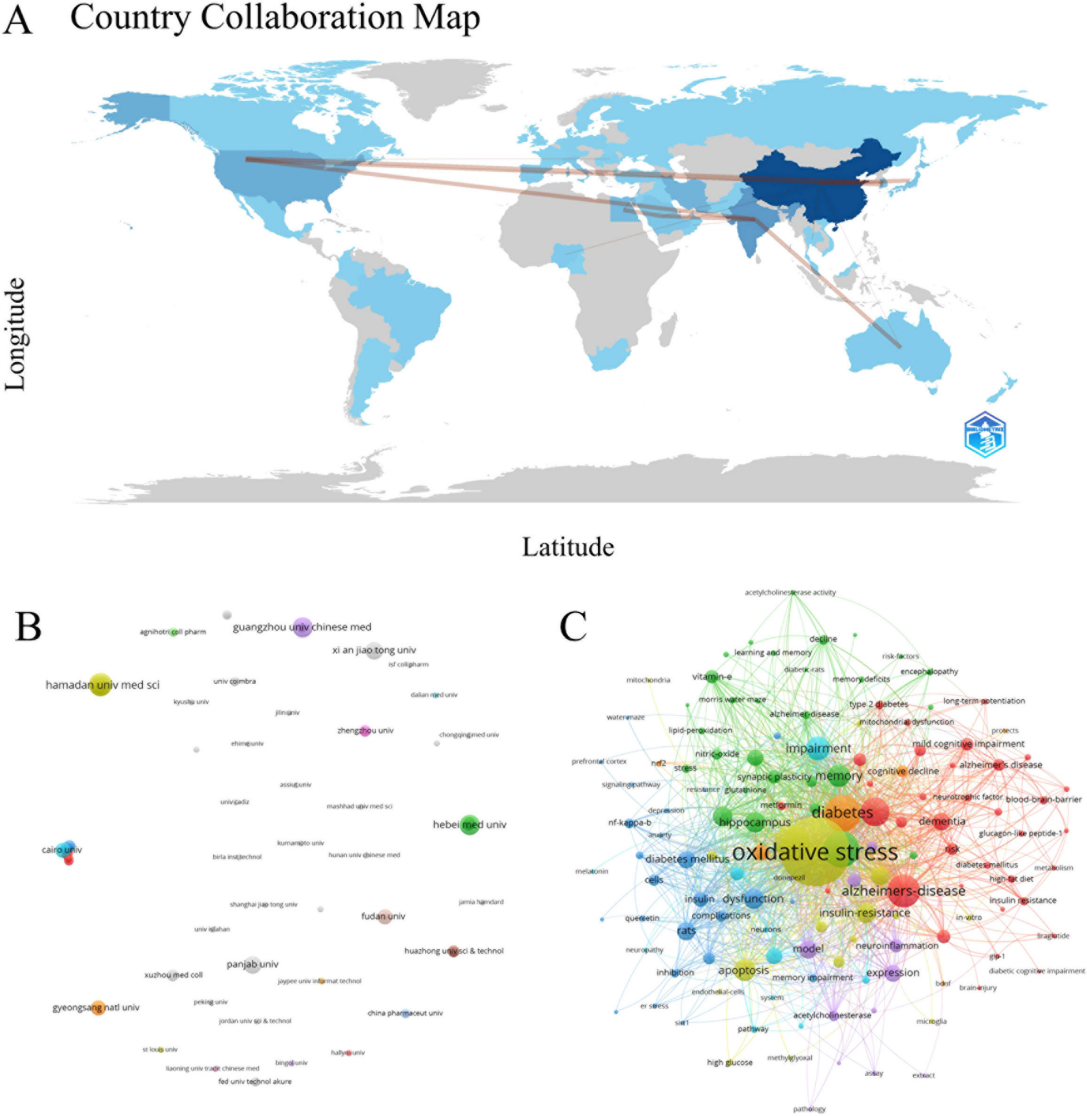
Country collaboration map of oxidative stress research in DCD **(A)**, institution co-occurrence network of oxidative stress research in DCD **(B)**, and keyword co-occurrence network of oxidative stress research in DCD **(C)**.

**Figure 4A** shows the partnership between countries, which demonstrates the existence of close cooperation between countries. A summary was provided for the top 10 countries based on their volume of articles (**Table 3**). China leads with 112 articles published, trailed by India and the United States with 46 and 30 articles, respectively. There are 8 countries where the number of published articles is greater than or equal to 10.

**Table 3:** Top 10 most relevant countries/regions for oxidative stress research in DCD.

Rank	Country	Document	Citations	Average Citation/Publication
1	Peoples R China	112	2356	21.04
2	India	46	1292	28.09
3	USA	30	1042	34.73
4	Iran	24	816	34.00
5	Egypt	18	366	20.33
6	South Korea	13	249	19.15
7	Japan	10	636	63.60
8	Saudi Arabia	10	73	7.30
9	Italy	8	200	25.00
10	Nigeria	7	120	17.14

### 2.6 Institution analysis

Through the analysis of institutions, the core research forces in the field of research can be mastered. A total of 452 institutions were found to be involved in the study through the analysis of VOSviewer software, and a co-occurrence network of research institutions was mapped by VOSviewer software (**Figure 4B**). **Table 4** lists 10 core institutions working in this area. Hamadan University of Medical Sciences has the highest number of publications, with 8 articles.

**Table 4:** Top 10 most relevant institutions for oxidative stress research in DCD.

Rank	Institution	Documents	Citations	Average Citation/Publication
1	HAMADAN UNIVERSITY OF MEDICAL SCIENCES	8	290	36.25
2	TEHRAN UNIVERSITY OF MEDICAL SCIENCES	7	251	35.86
3	GUANGZHOU UNIVERSITY OF CHINESE MEDICINE	7	223	31.86
4	HEBEI MEDICAL UNIVERSITY	7	141	20.14
5	PANJAB UNIVERSITY	6	424	70.67
6	XI’AN JIAOTONG UNIVERSITY	6	117	19.50
7	IRAN UNIVERSITY OF MEDICAL SCIENCES	5	123	24.60
8	SHAHED UNIVERSITY	5	249	49.80
9	CAIRO UNIVERSITY	5	20	4.00
10	FUDAN UNIVERSITY	5	171	34.20

### 2.7 Literature analysis

The 10 most cited documents on the role of oxidative stress in DCD are shown in **Table 5**. These articles offer guidance for researchers to delve deeper into the development of oxidative stress in DCD.

**Table 5:** Top 10 most-cited documents about the effect of oxidative stress on DCD.

Rank	Paper	DOI	Total Citations	TC per Year
1	LIN B, 2014, CARDIOVASC DIABETOL	10.1186/s12933-014-0148-1	274	27.40
2	KLEINRIDDERS A, 2015, P NATL ACAD SCI USA	10.1073/pnas.1500877112	262	29.11
3	MARKOWICZ-PIASECKA M, 2017, PHARM RES-DORDR	10.1007/s11095-017-2199-y	165	23.57
4	BHUTADA P, 2011, BEHAV BRAIN RES	10.1016/j.bbr.2011.01.022	156	12.00
5	PINTANA H, 2013, J ENDOCRINOL	10.1530/JOE-12-0521	143	13.00
6	KUHAD A, 2009, PHARMACOL BIOCHEM BE	10.1016/j.pbb.2008.12.012	137	9.13
7	KUHAD A, 2008, LIFE SCI	10.1016/j.lfs.2008.05.013	133	8.31
8	CARVALHO C, 2012, DIABETES	10.2337/db11-1186	121	10.08
9	BHUTADA P, 2010, NEUROBIOL LEARN MEM	10.1016/j.nlm.2010.06.008	120	8.57
10	KIMURA R, 2009, NEUROSCIENCE	10.1016/j.neuroscience.2009.05.025	105	7.00

### 2.8 Keyword analysis

Keywords can efficiently reflect the more concerned research content and the latest topics in a research field (17). After the partial selection of keywords by VOSviewer software, 296 nodes are formed in the keyword co-occurrence network (**Figure 4C**). In **Figure 4C**, the largest nodes are oxidative stress and diabetes, which are the same as the research theme, and high-frequency keywords such as Alzheimer’s disease (AD), cognitive decline, insulin resistance, and quercetin emerge, representing pivotal terms in this domain. Combined with the keywords and contents of the article, we found that the field mainly revolves around the role of oxidative stress in the development of diabetes to DCD and the antioxidant research of natural products (e.g. Quercetin) on DCD. This provides us with directions for a systematic review of the field next.

## 3 The pathogenesis of diabetes-induced cognitive dysfunction

**Figure 5:**
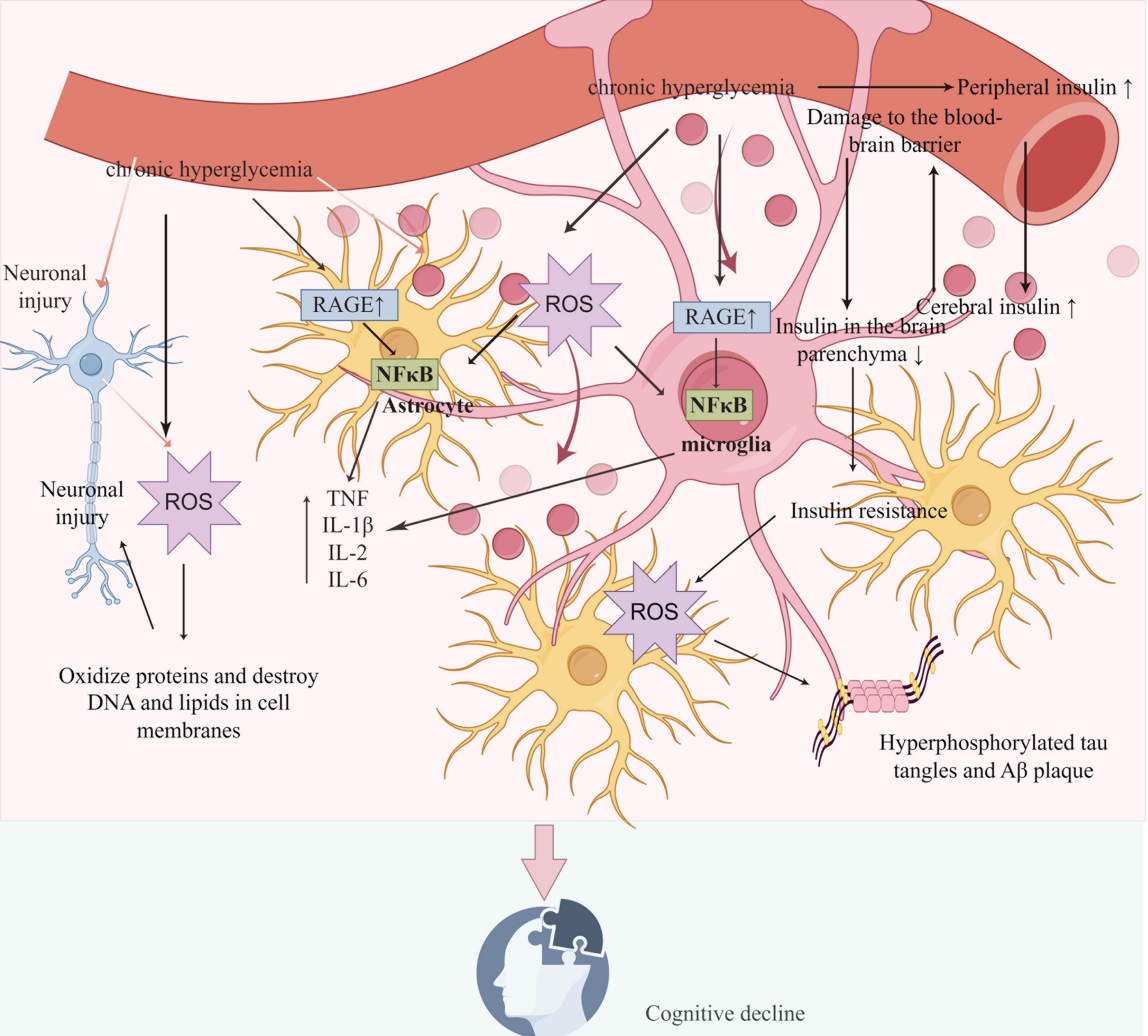
Pathogenesis of cognitive dysfunction caused by diabetes mellitus: The pathogenesis includes disorders of glucose and lipid metabolism, elevated levels of AGEs, oxidative stress, neuroinflammation, and damage of BBB by figdraw.

Type 2 diabetes mellitus (T2DM) is a significant risk factor for mild cognitive impairment and dementia (18). Diabetes can accelerate the progression of cognitive dysfunction. This impairment results from multiple interconnected factors, including disturbances in glucose and lipid metabolism, elevated levels of advanced glycation end-products (AGEs), oxidative stress, and neuroinflammation (19) (**Figure 5**).

T2DM is a multifactorial metabolic disease characterized by chronic hyperglycemia and dyslipidemia, which can induce long-term low-grade inflammation and oxidative stress, ultimately leading to extensive damage to target organs (11). Chronic hyperglycemia promotes the formation of AGEs (20), and the accumulation of AGEs increases the expression of their receptors (RAGE) in microglia, astrocytes (AST), and cerebral microvascular endothelial cells (ECs). This process induces oxidative stress, activates the nuclear factor-κB (NF-κB) pathway, and enhances the expression of intercellular adhesion molecules and pro-inflammatory factors, including tumor necrosis factor (TNF)-α, interleukin(IL)-6, and IL-1β (21). Chronic hyperglycemia leads to elevated levels of pro-inflammatory factors, resulting in prolonged activation of microglia. This shift transforms microglia from having neuroprotective effects to exerting neurotoxic effects, ultimately causing neuronal injury and cognitive decline (22). ROS, resulting from redox imbalance in patients with T2DM, are regarded as neurotoxic agents. These ROS can cause neuronal damage or death by oxidizing proteins and damaging deoxyribonucleic acid (DNA) and lipids in cell membranes, ultimately contributing to cognitive decline (10, 11). ROS can also induce insulin resistance (23), up-regulate inflammatory molecules, exacerbate inflammatory responses, and subsequently promote cognitive decline (12).

Insulin resistance in T2DM often progresses from prediabetes and impairs pancreatic buffering activity during hyperglycemia, and this insulin resistance can extend to the brain (24, 25). Most insulin in the brain originates from circulating pancreatic insulin, which crosses the blood-brain barrier (BBB) to enter the brain (26). In the early stages of insulin resistance, insulin levels in cerebrospinal fluid increased following a rise in peripheral insulin levels. Prolonged hyperinsulinemia can impair BBB function by either down-regulating insulin receptors on the BBB or altering its permeability, which subsequently reduces the transfer of insulin from peripheral circulation to the brain parenchyma. This change may significantly contribute to the development of insulin resistance in the brain (26–28). The pathological definition of AD is the presence of Aβ plaques and hyperphosphorylated tau tangles (29). Cerebral insulin resistance plays an important role in the occurrence and development of AD by increasing oxidative stress, stimulating the production of Aβ42 and phosphorylation of tau protein, which in turn impair mitochondrial function, cognitive function and memory (30).

In conclusion, there is a strong association between the pathophysiological mechanisms of T2DM and cognitive dysfunction. Cognitive dysfunction is a severe complication of T2DM, 2presenting significant challenges to both the healthcare system and socio-economic development.

## 4 Effect of oxidative stress on diabetic cognitive dysfunction

### 4.1 Overview of oxidative stress

Oxidative stress is a classical biological process and refers to a redox imbalance state in which oxidants such as ROS are produced in excess with impaired antioxidant defense systems in the body (31). Under physiological conditions, low levels of ROS and reactive nitrogen radical (RNS) are necessary to mediate cell signaling and maintain homeostasis (32), and there is a balance between O2^-^ production and antioxidant defense systems, and superoxide dismutase (SOD) is an important O2^-^ natural scavenger *in vivo* (33). Hydrogen peroxide (H_2_O_2_) can be further degraded into water and oxygen by catalase (CAT) or glutathione (GSH) redox systems; pathologically, once the redox balance between ROS production and antioxidant enzymes is compromised, a series of synergistic chain reactions will be initiated, resulting in more secondary ROS accumulation (34). Overproduction of ROS and RNS leads to the denaturation of proteins, lipids, DNA, and ribonucleic acid (RNA), which in turn gives rise to a sequence of pathological processes and accelerates cell aging and death (31).

### 4.2 The mechanism of oxidative stress in nerve injury

**Figure 6:**
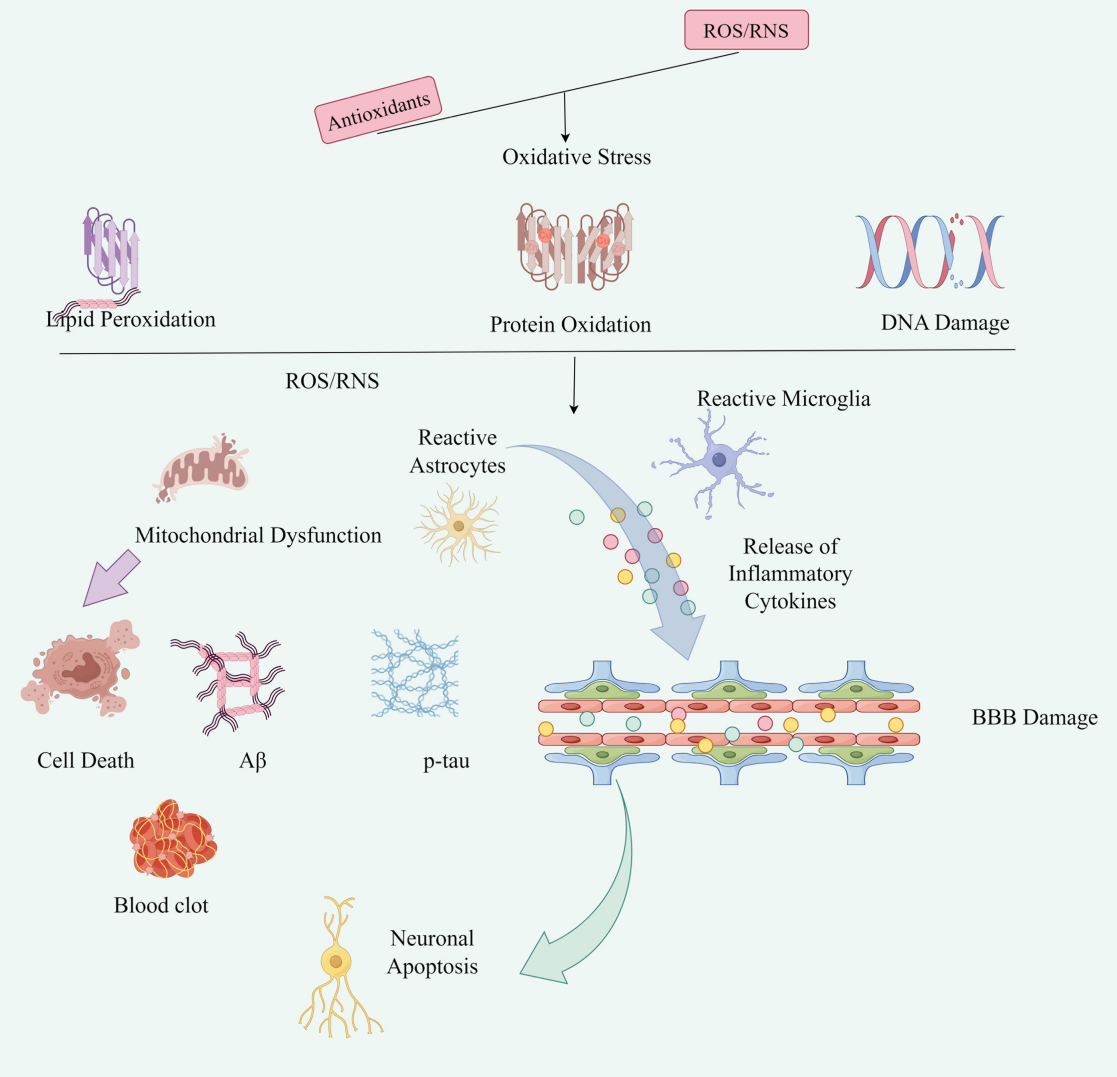
The mechanism of oxidative stress in nerve injury: Oxidative stress can lead to lipid peroxidation of nerve cell membrane, increase the permeability of cell membrane, and lead to imbalance of ion concentration and imbalance of internal and external environment of the cell. In addition, oxidative stress can directly damage biological molecules such as DNA and proteins of nerve cells, leading to cell dysfunction and apoptosis. At the same time, oxidative stress can trigger neuroinflammatory response, triggering the release of inflammatory cytokines, and further lead to nerve cell injury and apoptosis by figdraw.

An imbalance between oxidative and antioxidant processes leads to the accumulation of free radicals, such as ROS and RNS, which damages cells and tissues. Oxidation plays a significant role in nerve injury, with its mechanisms involving multiple factors (**Figure 6**).

1. Mechanism of oxidative stress in nerve injury
  1.1 Direct damage to nerve cells: ROS and RNS can directly damage biological macromolecules in nerve cells, including lipids, proteins, and nucleic acids. This damage leads to increased membrane permeability, intracellular calcium overload, mitochondrial dysfunction, and ultimately, nerve cell death (35, 36). Increased membrane permeability and intracellular calcium overload can activate a variety of calcium-dependent enzymes, phospholipase C and protease, which further aggravate nerve cells (37). Mitochondrial dysfunction can enhance ROS production and disrupt cellular energy metabolism, thus aggravating nerve cell injury (38).
  1.2 Induction of nerve cell apoptosis: Oxidative stress can activate intracellular apoptosis signaling pathways, such as mitochondrial pathway, death receptor pathway, etc., leading to nerve cell apoptosis (39).
  1.3 Promote inflammatory response: Oxidative stress can activate inflammatory cells and trigger the release of inflammatory factors, such as TNF-α and IL-1β, thereby exacerbating the inflammatory response following nerve injury (40).
  1.4 Affect the synthesis and release of neurotransmitters: Oxidative stress can affect the synthesis and release of neurotransmitters, resulting in an imbalance of neurotransmitters, which affects the transmission of nerve signals.
  1.5 Activation of redox signaling pathways: Oxidative stress can activate intracellular redox signaling pathways, including the nuclear factor E2-related factor 2 (Nrf2)/antioxidant response element (ARE) pathway (41) and the NF-κB pathway. Activation of these pathways regulates the expression of antioxidant enzymes and other antioxidants in cells, thereby reducing nerve cell damage caused by oxidative stress.
    In conclusion, oxidative stress plays a complex role in nerve injury, involving multiple mechanisms. It contributes to nerve cell damage by affecting cell membrane structure, damaging nucleic acids and proteins, initiating inflammatory responses, and altering synaptic transmission. Regulating oxidative stress balance, activating intracellular antioxidant defense systems, studying oxidative stress-related signaling pathways, and developing new antioxidant drugs have become key strategies for preventing and treating neurological diseases.

### 4.3 Oxidative stress promotes the progression of diabetes toward diabetic cognitive dysfunction

**Figure 7:**
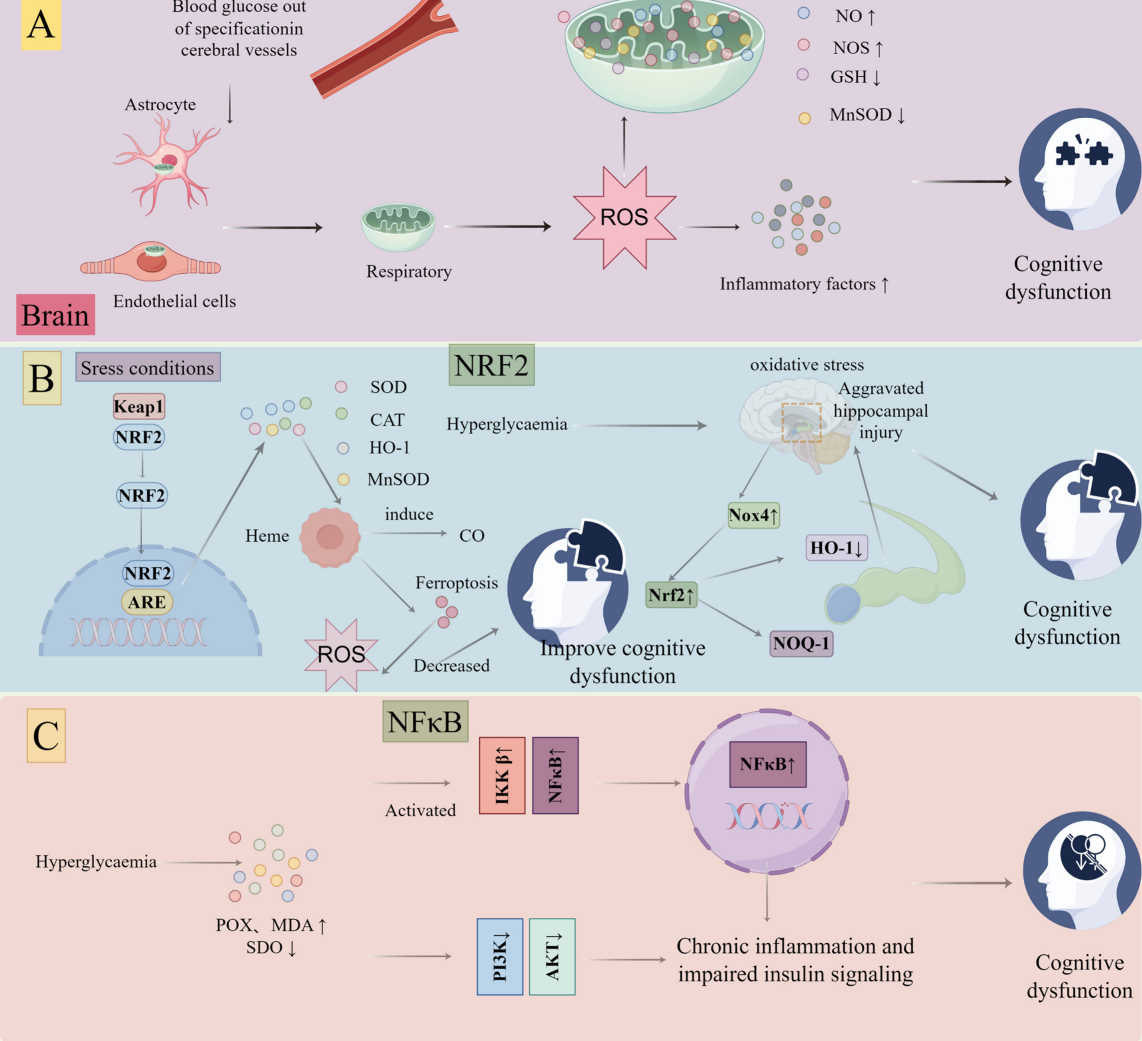
Oxidative stress promotes the progression of diabetes toward diabetic cognitive dysfunction by figdraw: Hyperglycemia increases mitochondrial respiration, promotes ROS levels, increases NO and NOS in brain mitochondria, and decreases MnSOD GSH-Px activity. Meanwhile, ROS enhances inflammatory factors, leading to cognitive dysfunction **(A)**. Hyperglycemia can promote Nrf2, NQO-1 and HO-1 downregulation, causing continuous oxidative stress, aggravating oxidative damage in the hippocampus, and causing cognitive dysfunction **(B)** and In the condition of hyperglycemia, POX and MDA increased and SOD activity decreased, which promoted the activation of IKKB/NF-κB and inhibited the activity of PI3K/PKB, further promoted chronic inflammation and impaired insulin signaling pathway, and led to cognitive dysfunction **(C)**.

Cognitive impairment is one of the complications of diabetes, characterized by structural and pathological changes in the brain, and increasing research indicates that oxidative stress plays a pivotal role in cognitive impairment (42). Hyperglycemia is one of the prominent features of diabetes and can increase mitochondrial respiration in endothelial cells and astrocytes, thereby promoting ROS production. With the increase of ROS level, mitochondrial nitric oxide (NO) level and nitric oxide synthase (NOS) expression increased, manganese superoxide dismutase (MnSOD) protein content and GSH peroxidase (GSH-Px) activity decreased in brain tissue (43). Elevated ROS can enhance the expression of inflammatory cytokines, and sustained inflammation can lead to cognitive dysfunction (44). On the other hand, ROS are also involved in regulating the transduction of various intracellular signaling pathways, such as Nrf2 -related signaling pathway and NF-κB, which activate neuroinflammation, immune mechanisms, and accelerate the pathological process of DCD (**Figure 7A**).

Nrf2, an important regulator of the antioxidant system, can coordinate a variety of cytoprotective factors to inhibit oxidative stress, and can also be involved in inhibiting oxidative stress by mediating related antioxidant pathways (Nrf2-ARE, Nrf2/HO-1, etc.) (45). Under stress conditions, the complex of Nrf2 and Kelch-like ECH-associated protein 1 (Keap1) is degraded. Nrf2 is aggregated in the cytoplasm and transported to the nucleus to bind with ARE and then initiates the transcription of antioxidant enzymes (SOD, CAT and MnSOD) (46). Heme-oxygenase (HO)-1 is one of the targets of Nrf2 and facilitates the degradation of heme, leading to the generation of carbon monoxide (CO) and the release of free iron, ultimately reducing the overall production of ROS (47). The mechanisms described above coordinate to form an antioxidant defense network, safeguarding the body against oxidative stress-induced damage. Liang et al. discovered, through experimental studies, that the expression of NADPH oxidase 4 (Nox4) was upregulated while the expression of Nrf2/HO-1 was downregulated in the hippocampus of diabetic rats (48). Subsequent studies revealed downregulation of Nrf2 expression and its downstream antioxidant genes (NQO-1 and HO-1) in both the cytoplasm and nucleus of hippocampal neurons in T2DM mice. It can cause continuous oxidative stress in a continuously high glucose environment, thus weakening the role of Nrf2, and further aggravating oxidative damage in the hippocampus, thus leading to cognitive impairment (49) (**Figure 7B**).

NF-κB is a member of the nuclear transcription factor protein family and serves as a crucial transcriptional regulator of immune and inflammatory responses. It represents a pivotal node and therapeutic target in pathophysiological mechanisms, including chronic inflammation, oxidative stress, and autoimmunity (50). The classical NF-κB is activated in response to various external stimuli involving inflammation, immune response, cell proliferation, differentiation, and survival. ROS may be a general modulator of NF-κB signaling pathway activation (51). The production of oxygen free radicals induced by hyperglycemia can stimulate the production of pro-inflammatory cytokines and enhance NF-κB expression, contributing to diabetes-related cognitive deficits (52). The study revealed that diabetes markedly elevated peroxide products (POX) and malondialdehyde (MDA) while reducing SOD activity in the hippocampus. Subsequently, lipid peroxidation promoted the activation of IκB kinase-β (IKKβ)/NF-κB, which further mediated chronic inflammation and impaired insulin signaling by inhibiting insulin-stimulated phosphatidylinositol 3-kinase (PI3K) and protein kinase B (PKB) activities (53). This suggests that effective inhibition of oxidative stress-mediated transduction of the NF-κB signaling pathway is an important means to prevent and treat DCD (**Figure 7C**).

## 5 The antioxidant research of natural products on diabetic cognitive dysfunction

As DCD has gained increasing attention from researchers, there is increasing interest in natural products. More and more researchers began to study the antioxidant effects of natural products on DCD, and a multitude of experiments were conducted *in vivo* and *in vitro* (**Table 6**).

**Table 6:** The antioxidant research of nature products on DCD.

Rank	Natural product	Cell/Animal Model	Dosage	Dosing method	BBB penetration ability	Indexes	Ref
1	Andrographis paniculata	STZ(Wistar)	100mg/kg 50mg/kg	oral administration intraperitoneal injection		↑:SOD ↓:MDA	(54)
2	Asiaticoside	STZ(SD)	20,40mg/kg	oral administration	yes, apparent permeability: 70.61 ± 6.60	↑:GSH, SOD, CAT	(55)
		SH-SY5H cell	1µmol/l			↓:ROS	
3	Astragaloside IV	STZ(C57BL/6J)	40mg/kg	oral administration		↑:SOD ↓:MDA, ROS	(56)
		STZ(SD)	40,80mg/kg	oral administration		↑:SOD ↓:MDA	(57)
4	Berberine	STZ(Wistar)	25,50,100mg/kg	oral administration	yes	↑:GSH ↓:MDA	(58)
		STZ(Wistar)	50,100mg/kg	oral administration		↑:SOD ↓:MDA, NIT	(59)
5	Betaine	STZ(Wistar)	25,50,100 mg/kg	intraperitoneal injection		↑:SOD ↓:MDA	(60)
6	Calycosin	STZ(SD)	20mg/kg	intraperitoneal injection		↑:SOD, GSH-Px ↓:MDA	(61)
7	Crocin	STZ(Wistar)	15,30,60 mg/kg	intraperitoneal injection		↑:TBARS	(62)
8	Diosgenin	STZ(Wistar)	40mg/kg	oral administration		↑:SOD, GSH ↓:MDA	(63)
9	Esculin	STZ(C57BL/6J)	5,10,20mg/kg	intravenous drip		↑:SOD ↓:MDA	(64)
10	Flos Puerariae	STZ(C57BL/6J)	50,100,200mg/kg	oral administration		↑:SOD, GSH-Px, CAT ↓:MDA	(65)
11	Genistein	STZ (Swiss albino male mice)	2.5,5.0,10.0mg/kg	intraperitoneal injection		↑:GSH ↓:MDA	(66)
		STZ (Swiss albino male mice)	2.5,5.0,10.0mg/kg	intraperitoneal injection		↑:GSH ↓:TBARS	(67)
12	Ginsenoside Re	STZ(SD)	40mg/kg	oral administration	yes, hypodermic injection Ginsenoside Re peaks in the cerebrospinal fluid after 60 minutes	↑:GSH ↓:MDA	(68)
13	H. zeylanica	STZ(Wistar)	300,400mg/kg	oral administration		↑:SOD, CAT, GSHx ↓:MDA	(69)
14	Huperzine A	STZ(Wistar)	0.05,0.1mg/kg	intraperitoneal injection	yes, tail vein injection brain 0.5mg/kg: 37.98ng/mg	↑:SOD,GSH-Px, CAT ↓:MDA	(70)
15	Hyperoside	STZ(Wistar)	50,200,400mg/kg	oral administration	yes, tail vein injection 140mg/kg: 240.183 ± 14.457mg/ml	↑:SOD, GSH-Px, CAT, TAC ↓:MDA, iNOS	(71)
16	Luteolin	STZ(SD)	50,100mg/kg	oral administration		↑:GSH, SOD, CAT ↓:MDA	(72)
17	Lycopene	STZ(Wistar)	1,2,4 mg/kg	oral administration	yes, oral brain lycopene dissolved in olive oil: 20.62 ± 3.39 ng/ml lycopene-loaded microemulsion: 143.86 ± 15.27 ng/ml	↑:SOD, CAT ↓:TBARS, NO	(73)
18	Mangiferin	STZ(SD)	15,30,60mg/kg	oral administration		↑:SOD, GSH ↓:MDA	(74)
19	Naringin	STZ(Wistar)	100mg/kg	oral administration	yes, femoral vein brain 120 mg/kg: 0.64 ± 0.18µg/ml	↑:SOD ↓:MDA	(75)
20	Neferine	db/db mice	25,50mg/kg	oral administration		↑:SOD, GSH-Px ↓:MDA	(76)
21	Puerarin	STZ(Wistar)	100mg/kg	oral administration	yes, intraperitoneal injection hippocampus (µg/mL) 80 mg/kg 3.35 ± 0.55 40 mg/kg 2.09 ± 0.31 20 mg/kg 1.58 ± 0.24 cerebral cortex (µg/mL) 80 mg/kg 4.48 ± 0.86 40 mg/kg 3.56 ± 0.61 20 mg/kg 1.73 ± 0.24 striatum (µg/mL) 80 mg/kg 1.93 ± 0.37 40 mg/kg 1.55 ± 0.17 20 mg/kg 1.03 ± 0.22	↑:SOD ↓:MDA	(77)
22	Quercetin	STZ(Wistar)	5, 25, 50mg/kg	oral administration	yes, 100 mg/kg oral brain 842.1 ± 508.4 mg/L	↓:MDA	(78)
		db/db mice	35, 70mg/kg	oral administration		↑:SOD, CAT, GSH-PX ↓:MDA	(79)
23	Resveratrol	STZ(SD)	10,20mg/kg	oral administration	yes, oral brain 15.16mg/kg:2mg/kg	↑:GSH, SOD, CAT ↓:MDA	(80)
24	Saffron	STZ(Wistar)	20,40,80mg/kg	intraperitoneal injection		↑:SOD, GSH, CAT ↓:iNOS	(81)
25	Safranal	STZ(Wistar)	0.025,0.1,0.4mg/kg	intraperitoneal injection		↑:SOD ↓:MDA	(82)
26	Salidroside	STZ (83)	200mg/kg	oral administration	yes, intravenous brain 15mg/kg: 0.1ug/g	↑:GSH-Px, GSH, SOD ↓:MDA	(42)
27	Securidaca inappendiculat	STZ(Wistar)	50,100,200mg/kg	oral administration		↑:GPX, GSH, CAT, SOD ↓:MDA	(84)
28	Sesamin	STZ(Wistar)	30mg/kg	oral administration		↑:SOD, CAT, GSH-Px ↓:MDA	(10)
29	sesamol	STZ(Wistar)	2,4,8 mg/kg	oral administration	yes, 4mg/kg brain intravenous: 272.45 ug/ml oral: 15.34 ug/ml	↑:GSH, SOD, CAT ↓:TBARS	(85)
30	Tanshinone IIA	STZ(Wistar)	2,4 mg/kg	intraperitoneal injection	yes, 5mg/kg intravenous Brain: 1.8ug/g	↑:SOD ↓:MDA	(86)
31	Tetramethylpyrazine	STZ (SD)	20,40,80mg/kg	intraperitoneal injection	yes, 10mg/kg intracenous brain: 184.6 ± 15.6 ug/ml	↑:SOD, CAT, GSH ↓:MDA, PCO	(87)

↑, Up; ↓, Down.

### 5.1 Polyphenols

Polyphenolic compounds are secondary metabolites produced by plant tissues, which are widely present in plant foods and can be obtained from various fruits, vegetables, teas, coffee, alcoholic beverages, and whole wheat foods.

#### 5.1.1 Resveratrol

Resveratrol, a natural antioxidant with a relative molecular weight of 228, is present in various fruits, vegetables, and traditional Chinese medicine (88). Resveratrol crosses the BBB in detectable amounts in the brain, but at very low concentrations because oral resveratrol has poor bioavailability (89, 90) (see **Table 6**). Multiple studies have indicated that resveratrol exhibits neuroprotective properties, mitigating memory deficits caused by oxidative damage and ameliorating cognitive decline in diabetic rat models (91). The findings indicated that chronic supplementation of resveratrol markedly inhibited the increase in MDA levels and significantly ameliorated the decrease in reduced GSH levels, as well as the reduced activity of SOD and CAT enzymes in the hippocampus of diabetic rats (80). 3-Nitrotyrosine (3-NT), 8-hydroxy-2 deoxyguanosine (8-OHdG), and MDA levels were able to assess oxidative stress levels, and Wang et al. found that resveratrol significantly prevented hippocampus 3-NT accumulation and 8-OHdG overexpression induced by T2DM, and reduced levels of MDA, a lipid peroxidation product (49). These studies indicate that resveratrol treatment may ameliorate diabetic cognitive impairment through its antioxidation, and further research has found that the antioxidant effect of resveratrol may be due to silent regulatory protein (SIRT) 2 regulation (92).

#### 5.1.2 Quercetin

Quercetin, a natural plant compound with strong antioxidant activity, is abundantly found in kale, onion, berries, apples, red grapes, broccoli, and cherries as well as tea and red wine, and has neuroprotective, anticancer, antidiabetic, anti-inflammatory, and free radical scavenging abilities. Animal studies have demonstrated a significant increase in brain MDA content in diabetic rats, while treatment with quercetin resulted in decreased MDA levels in rat brains (78). Another study found that db/db mice had elevated MDA levels and decreased SOD, CAT, and GSH-PX activities in the brain, while quercetin significantly reduced MDA levels, increased SOD, CAT, and GSH-PX activities and ameliorated oxidative stress (79). Further studies have shown that pretreatment with quercetin and nano-quercetin could effectively regulate antioxidant levels, by down-regulating NF-κB, decreasing the levels of MDA and NO, as well as increasing brain serotonin levels in the rat brain and reducing neurodegeneration and cognitive deficits, and may protect against antioxidant stress cognitive deficits through modulating the Nrf2/HO-1/Keap-1 pathway (93) It was found that orally administered 100 mg/kg quercetin was detectable in the rat brain, suggesting that quercetin was able to cross the BBB (see **Table 6**) (94).

#### 5.1.3 Sesamol and sesamin

Sesamol is one of the secondary lignans isolated from sesame seeds and is also a natural phenolic compound with a very strong antioxidant capacity (95). Sesamol is well absorbed in the whole gastrointestinal tract and has a bioavailability of 95.61% by mouth. It has good biological characteristics and can easily pass the BBB (96)(see **Table 6**). Sesamol improved learning and memory behavior in streptozotocin (STZ) rats by reducing levels of thiobarbituric acid reactive substances (TBARS) in various brain regions and restoring GSH, SOD, and CAT levels to normal in the cerebral cortex and hippocampus (85).

Sesamin, a prominent lignan extracted from sesame seeds and their oils, exhibited potent hypoglycemic effects (97). Animal studies have found that chronic treatment with sesamin can increase the activity of CAT, GSH-Px, and SOD, inhibit the elevation of MDA level, and improve the oxidation state of the hippocampus of STZ rats, indicating that sesamin can scavenge free radicals. Thus, the improvement of sesamin on DCD may be due in part to its protective effect against diabetes-induced oxidative stress in the hippocampus (10).

#### 5.1.4 Mangiferin

Mangiferin (MF) is the primary oral kaempferoside found in Anemarrhena asphodeloides, exhibiting anti-inflammatory, antioxidant, anti-diabetic, and other beneficial biological activities (98). The results of animal studies found that MF could reduce the MDA level in hippocampal tissue and increase the serum levels of GSH and SOD in diabetic rats, which in turn improved the learning memory ability of diabetic rats (74). Cellular-level studies demonstrated that MF effectively restored SOD activity and GSH levels to near-normal levels in hippocampal neurons cultured in a high-glucose environment (99).

#### 5.1.5 Hyperoside

Hyperoside (HYP) is a flavonoid found in various medicinal plants such as Hypericum, Labiatae, etc. HYP can successfully reach the brain through the BBB, and the metabolic process is rapid, reaching a peak value about 0.5 h after entering the brain, and the basic metabolism is complete within 3 h (100) (see **Table 6**). HYP is recognized as a natural anti-inflammatory agent and a scavenger of ROS. Scholars have increasingly focused on HYP as a monomer in traditional Chinese medicine due to its diverse pharmacological effects, including antioxidative stress properties, enhancement of cardiovascular function, and regulation of endocrine function. HYP has an antioxidant capacity against oxidative stress in neurons and has been shown to enhance cognitive and retention abilities in various models (101–103). In addition, it has been found that HYP significantly inhibited the oxidative damage observed in the brain of T2DM rats, such as significantly decreasing the concentrations of MDA and inducible nitric oxide synthase (iNOS) and correspondingly increasing the activities of GSH-Px, CAT, SOD and the tricarboxylic acid cycle (TAC), thereby improving cognitive impairment in T2DM rats (71).

#### 5.1.6 Genistein

Genistein is a class of isoflavonoid compounds present in plant species such as soybean root and kudzu root, with pharmacological effects of antioxidant, improvement of fat accumulation, and prevention of cardiovascular disease. Animal studies have demonstrated that genistein regulates cholinergic neurotransmission, restores antioxidant balance (MDA and GSH levels return to normal), reduces neuroinflammatory markers and nitrite levels in the cerebral cortex and hippocampus, leading to improved cognition (66). Another study indicated that genistein might safeguard against diabetes-associated memory deficits by normalizing TBARS levels in the brains of diabetic mice with cerebral ischemia-reperfusion and restoring GSH levels to those of control values (67).

#### 5.1.7 Calycosin

Calycosin is a flavonoid of Astragalus membranaceus, which is one of the effective active components of Astragalus membranaceus and has anti-inflammatory, anti-tumor, hypoglycemic and cardioprotective properties (104, 105). It is reported that calycosin can significantly improve neurological deficits and infarct size in experimental models of cerebral ischemia-reperfusion injury (106). Animal studies have found that calycosin can improve spatial studying and memory and up-regulate the expression of synaptic proteins and brain-derived neurotrophic factor (BDNF) in the hippocampus of diabetic rats, and this effect may be associated with up-regulation of SOD, GSH-Px levels and down-regulation of MDA levels through the regulation of PI3K/Akt/glycogen synthase kinase 3β (GSK-3β) pathway (61).

#### 5.1.8 Puerarin

Puerarin, an active ingredient derived from the kudzu vine root in traditional Chinese medicine, exhibits antidiabetic properties. On the one hand, Puerarin can increase serum insulin and lower blood sugar levels, thereby reducing the occurrence of diabetes caused by STZ. On the other hand, puerarin has neuroprotective effects (107). The results of the basic research showed that Puerarin was able to effectively improve the cognitive dysfunction induced by STZ in diabetic rats, which may be due to Puerarin’s ability to significantly increase the SOD activity, reduce the level of MDA, and regulate the levels of IL-1β, IL-6, and TNF-α in hippocampal tissue of diabetic rats (77). Puerarin can be detected in the hippocampus, cortex and basal ganglia of rats after intrabitoneal injection, which indicates that Puerarin can pass the BBB and play a protective role (see **Table 6**) (108).

#### 5.1.9 Naringin

Naringin, a natural flavonoid glycoside found in grapefruit and oranges, is less effective orally, but it easily penetrates the BBB (see **Table 6**) (109) and has anti-apoptotic, antioxidant, anti-inflammatory, lipid-lowering and insulin-like properties. Studies have found that naringin significantly inhibits oxidative stress by increasing SOD level and decreasing MDA content, thus enhancing the cognitive function of diabetic rats (75).

#### 5.1.10 Luteolin

Luteolin, a natural flavonoid abundant in anti-inflammatory plant sources like celery, chrysanthemum, and oranges, exhibits anti-apoptotic, anti-allergic, and anti-cancer properties. It serves as both an antioxidant and a pro-oxidant in biochemistry (110, 111). The findings from animal studies demonstrate that prolonged use of luteolin can mitigate the elevation of MDA levels in the cerebrum of diabetic rats. Additionally, it reduces the activities of GSH, SOD, CAT, and cholinesterase in the cerebrum, thereby ameliorating neuronal damage and enhancing cognitive abilities in diabetic rats (72).

#### 5.1.11 Lycopene

Lycopene, a carotenoid present in plant foods like tomatoes, guavas, and carrots, acts as a potent natural pigment with antioxidant properties (112) It also exhibits neuroprotective, anti-inflammatory, cognitive enhancement, and cholesterol-lowering effects. In addition, lycopene can enter the central nervous system through the blood-brain barrier, lycopene dissolved in olive oil and lycopene-loaded microemulsion improve oral bioavailability and have good brain targeting ability (113) (see **Table 6**). It was found that chronic lycopene could significantly reduce the levels of TBARS and total NO and increase the enzymatic activities of protein thiol, SOD, and CAT in the hippocampus and cortex of STZ rats, and also reduce the expression of serum inflammatory factors in STZ rats, thereby improving cognitive impairment (73).

### 5.2 Alkaloids

#### 5.2.1 Berberine

Berberine (BBR), an alkaloid widely found in the roots (114), rhizomes, stems, or barks of several traditional herbs, has been reported to possess anxiolytic, antidepressant, and antiemetic effects (115). Many clinical and preclinical studies have proven the beneficial effects of berberine in diabetes mellitus, mainly due to its ability to enhance insulin expression and potential antioxidant effects, as well as improving spatial reminiscence impairment in diabetic rats (116). Animal studies have demonstrated that berberine reduces MDA and nitrite (NIT) levels in the hippocampal tissues of STZ rats while increasing SOD activity, thereby exerting neuroprotection (59). Another animal experimental study discovered that chronic treatment with berberine significantly and dose-dependently decreased levels of oxidative stress markers (MDA, GSH), thereby ameliorating cognitive deficits and cholinergic dysfunction in diabetic rats (58). A study using fluorescence-labeled berberine derivative BBR to study BBB permeability in cell models of Parkinsonism and Zebrafish Parkinsonism models found that BBR can easily penetrate BBB and function in the brains of Zebrafish with Parkinsonism (117) (see **Table 6**).

#### 5.2.2 Tetramethylpyrazine

Tetramethylpyrazine (TMP) is an active compound extracted from the rhizome of the Chinese herb Ligusticum chuanxiong, which has strong antioxidant properties and improvement of brain circulation and other pharmacological effects. TMP is reported to have appreciable BBB penetrability (118)(see **Table 6**). It is well-known that TMP is a free radical scavenger that can assist reduce oxidative stress, suppress inflammation, activate SIRT1, and have significant effects on cognitive function (87). Animal studies have found that TMP can alleviate oxidative stress and restore antioxidant condition in the hippocampus of DM rats by decreasing MDA levels and protein carbonylation (PCO) levels, as well as increasing CAT, GSH-Px, and SOD concentrations, and ultimately improve cognitive impairment in diabetic rats (87). Another study found that TMP via intracranial administration was able to prevent the cognitive behavioral phenotype of diabetes by modulating the SIRT1/Nrf2/HO-1 pathway, increasing the concentrations of GSH and SOD, as well as decreasing the content of mitochondrial DNA in the hippocampus of STZ-AD mice (119).

#### 5.2.3 Betaine

Betaine is a stable natural compound known for its protective effects on diabetes (120). Huang et al. showed that betaine could reduce MDA levels and increase SOD levels in the hippocampus of diabetic rats, which may be achieved by modulating the PI3K/Akt pathway and improve the oxidative stress state, which ultimately leads to the improvement of diabetic cognitive impairment (60).

#### 5.2.4 Neferine

Neferine is a dibenzyl tetrahydroisoquinoline alkaloid isolated from lotus seed and has biological activities such as anti-hypertension, anti-arrhythmia, and anti-oxidation (121). Neferine was found to ameliorate cognitive impairment in db/db mice, potentially by reducing MDA concentrations and enhancing GSH-Px and SOD levels in the hippocampus, thereby alleviating oxidative stress and restoring antioxidant defenses (76).

#### 5.2.5 Huperzine A

Huperzine A (HupA) is a potent and reversible cholinesterase inhibitor characterized by its high-fat solubility and small molecular weight. This property facilitates its penetration of the blood-brain barrier (122) (see **Table 6**), enabling it to augment the acetylcholine content in the synaptic gap, compensating for the deficient acetylcholine neurotransmitter. Hup A exhibits antioxidative properties, safeguards against cerebral hypoxia and ischemic injury, and enhances learning and memory (123). Hup A was observed to ameliorate diabetic cognitive impairment by modulating BDNF levels, oxidative stress, inflammation, and apoptosis in the brains of STZ rats (70).

### 5.3 Saponins

#### 5.3.1 Astragaloside IV

Astragaloside IV (AS-IV) is a tetracyclic triterpenoid saponin known for its antitumor effects and protective properties on the liver and kidneys. It is utilized in the treatment of cancer, vascular diseases, and diabetic complications (124). AS-IV treatment was found to significantly ameliorate the increase of ROS in the cortex and hippocampal CA1 of T2DM mice, decrease MDA levels, increase SOD activity in T2DM mice, and the Nrf2/Keap1/HO1/NQO1 pathway is the main pathway by which AS-IV reduces oxidative stress after T2DM, which in turn reduces cognitive impairment in T2DM mice (56). A separate study demonstrated that various doses of AS-IV mitigated nerve injury, elevated SOD activity, reduced MDA levels, and ameliorated insulin resistance in DCD rats. These effects were associated with increased expression of growth hormone secretagogue receptor (GHS-R1α) and the upregulation of phosphorylated AMP-activated protein kinase (p-AMPK)/peroxisome proliferators-activated receptor γ coactivator 1 α (PGC-1α)/uncoupling protein 2 (UCP2) (57).

#### 5.3.2 Diosgenin

Diosgenin is a kind of natural steroidal saponin widely found in the roots and stems of Dioscorea spp, such as Andrographis paniculata, Dioscorea peltata and Dioscorea spp. It exhibits efficacy in hypoglycemia, anti-inflammatory action, antioxidant properties, regulation of lipid metabolism, and prevention of cardiovascular diseases, among others (125). Diosgenin has been shown to alleviate neuropathic pain and exhibit anti-hyperglycemic effects through the reduction of oxidative stress and inflammation. Additionally, it can ameliorate oxidative damage and mitigate cognitive decline in aging mice following the D-galactose challenge (126). Long-term administration of diosgenin was found to decrease MDA levels and increase SOD and GSH activities in the hippocampal tissues of STZ-induced diabetic animals. Additionally, it mitigated learning and memory impairment by regulating NF-κB/toll-like receptors 4 (TLR4)/Nrf2 pathways, reducing astrogliosis biomarkers, restoring cholinergic function, and modulating the inflammatory response (63).

#### 5.3.3 Ginsenoside Re

Ginsenoside Re, an important active component in ginseng, is a tetracyclic triterpenoid derivative known for its significant hypoglycemic effects (127). It was found that there was a peak concentration (C_max_) in the cerebrospinal fluid of rats 60 minutes after subcutaneous injection of Ginsenosides Re (128) (see **Table 6**). Ginsenoside Re has been identified as playing a crucial role in the treatment and prevention of diabetes-related cognitive decline. It achieves this by reducing MDA levels and boosting the activity of the antioxidant GSH in the serum and brain of STZ rats (68).

#### 5.3.4 Asiaticoside

Asiaticoside is a white needle-like crystal extracted from Centella asiatica, and its main component is triterpenoid saponins (129). Sun et al. demonstrated that asiaticoside exhibits protective effects against ischemia-hypoxia-induced neuronal cell injury, alleviates diabetic microangiopathy, and facilitates nerve regeneration (130, 131). Asiaticoside was found to restore synaptic features and enhance learning and memory abilities in diabetic rats. This effect may be attributed to its antioxidative properties, including the increase in SOD, CAT activity, and GSH levels in the hippocampus of diabetic rats, and the inhibition of ROS production in SH-SY5H cells treated with high glucose. Additionally, it exhibits anti-inflammatory effects and regulates the PI3K/Akt/GSK-3 pathway (55). An *in vitro* blood-brain barrier model study in primary pig brain endothelial cells (PBECs) showed that asiaticoside can cross the blood-brain barrier with an apparent permeability of 70.61 ± 6.60×10^−6^ cm/s (132) (see **Table 6**).

#### 5.3.5 Salidroside

Salidroside is a natural plant saponin isolated from the rhizome of the Tibetan medicine Rhodiola rosea and a wide range of pharmacological properties such as anti-hypoxia, anti-oxidation, anti-inflammation, anti-fatigue, anti-aging and immune regulation, and can play a neuroprotective role through the blood-brain barrier (133). Research reports suggest that Salidroside can elevate SOD, GSH, and GSH-Px levels while reducing MDA concentration, thereby potentially mitigating cognitive impairment induced by diabetes (42).

### 5.4 Others

#### 5.4.1 Esculin

Esculin, a coumarin compound, is extracted from the dried bark of Oleaceae plants, including Caulis Carpini and Caulis cuspidate. It serves as one of the principal active constituents of Cortex Fraxini. Esculin was discovered to ameliorate cognitive impairment via the mitogen-activated protein kinase(MAPK) signaling pathway and exhibited antioxidative effects by reversing the inhibition of SOD activity and increasing MDA levels in a diabetic nephropathy mouse model, along with anti-inflammatory effects (64).

#### 5.4.2 Crocus sativus L

Crocus sativus L., a member of the iris family, is the foremost among the 39 traditional Chinese medicines prioritized for development in China, used to treat a range of ailments such as cardiovascular, mental, and digestive disorders (134, 135). Experimental studies have indicated that saffron aqueous extract may improve memory deficits in diabetic rats by affecting GSH, SOD, and CAT levels, as well as iNOS levels in the hippocampus (81).

Safranal, a principal chemical constituent of saffron, has garnered attention for its antioxidant, oxidative stress inhibitory, anti-inflammatory, and anti-apoptotic properties. Existing studies have demonstrated that safranal effectively protects tissues including the brain, myocardium, skeletal muscle, and retina from oxidative damage (136). Safranal treatment alleviated hyperglycemia, learning and memory deficits, hippocampal neuronal loss, and imbalances in MDA, TNF-α, and caspase-3 levels, while also restoring SOD activity in hippocampal tissue (82).

Crocin, the principal bioactive compound in saffron, exhibits a range of effects including anti-inflammatory, antihypertensive, hypoglycemic, hypolipidemic, antioxidant, and antidepressant properties (137). Crocin ameliorates memory deficits across various models. Studies suggest that crocin may directly or indirectly decrease TBARS levels by alleviating hyperglycemia, thus ameliorating memory dysfunction induced by STZ (62).

#### 5.4.3 Tanshinone IIA

Tanshinone IIA (Tan IIA) is a representative fat-soluble component of salvia miltiorrhoea, which has a variety of physiological and pharmacological effects such as natural antioxidant, anti-tumor, anti-inflammatory, antibacterial, etc. It has a protective effect on cardiovascular diseases (138) and can protect nerves through the BBB (139) (see **Table 6**). Animal studies suggest that Tan IIA may reduce hippocampal oxidative stress by reducing MDA levels and increasing SOD activity, thereby enhancing learning and memory in diabetic rats (86).

#### 5.4.4 Radix Puerariar

Radix Puerariar is the hypertrophic tuberous root of the legume Pueraria lobata or Pueraria lobata, which contains a variety of active ingredients, mainly isoflavones, especially puerarin. Treatment with Radix Puerariar extract markedly normalized metabolic disorders and improved cognitive deficits in STZ-induced experimental diabetic mice. These effects may be attributed to the suppression of oxidative stress (by increasing SOD, CAT, and GSH-Px activities and reducing MDA levels in the cortex and hippocampus of diabetic mice) and regulating acetylcholinesterase activity in the brain (65).

#### 5.4.5 Securidaca inappendiculata

Securidaca inappendiculata (SI) is a plant belonging to the genus Securidaca in the family Polygalaceae. SI administration markedly enhances GPX, GSH, CAT, and SOD activities while reducing MDA levels, thereby alleviating oxidative stress. Consequently, SI significantly ameliorates memory impairment, cognitive dysfunction, and hyperalgesia in hyperglycemia-induced diabetic rats. These effects may be attributed to the modulation of the p38 MAPK/Nrf2/HO-1 pathway (84).

#### 5.4.6 Hydrolea zeylanica

Hydrolea zeylanica, an aquatic perennial creeping plant often included in diets, is abundant in nutrients and aids in regulating blood glucose levels. Administration of Hydrolea zeylanica extract at a dose of 400 mg/kg significantly decreased blood glucose levels and elevated serum insulin levels in diabetic encephalopathy rats induced by STZ. Furthermore, it enhanced antioxidant activity (MDA, SOD, CAT, and GSH) in brain tissue, leading to improvements in cognitive function (69).

#### 5.4.7 Andrographis paniculata

Andrographis paniculata, a commonly used traditional medicine in Chinese folk medicine, is a plant resource with a wide range of development prospects and has antipyretic, anti-inflammatory, immunomodulatory effects, and treatment of cardiovascular diseases (140). Andrographis paniculata leaves extract was shown to decrease MDA levels and increase SOD levels in brain tissue, showing significant cerebral protection, and pro-neural activity in normal and T2DM rats (54).

## 6 Conclusions and future perspectives

This paper introduces a novel approach to investigate the impact of oxidative stress on DCD, aiming to identify a new research direction via scientometric analysis. Based on the results of scientometric analysis, this paper reviews the effects of oxidative stress on DCD and the antioxidant research of natural products on DCD.

Oxidative stress plays a crucial role in the development of DCD, engaging multiple intricate signaling pathways, such as the Nrf2 and NFκB pathways. Currently, several natural products possess antioxidant and antihyperglycemic properties that hold the potential for preventing or impeding the progression of DCD. Despite the relatively clear efficacy and targets of natural products, there remains insufficient research on their medicinal properties. While the antioxidant effects of natural products in ameliorating DCD have been extensively investigated in preclinical studies, their evaluation in clinical trials is currently infrequent.

The low bioavailability and absorption rate of antioxidant natural products currently limit their clinical application and further improvement of their absorption and bioavailability is required and their potential side effects are explored in clinical trials. Improved bioavailability and standardized dosages of crude extracts or purified bioactive compounds are anticipated. Hopefully, in conjunction with existing treatments, antioxidant natural products will substantially alleviate the burden of DCD.
